# Induction of Macrophage M2b/c Polarization by Adipose Tissue-Derived Mesenchymal Stem Cells

**DOI:** 10.1155/2019/7059680

**Published:** 2019-06-19

**Authors:** Meng Sun, Linxiao Sun, Chongchu Huang, Bi-cheng Chen, Zhenxu Zhou

**Affiliations:** Key Laboratory of Diagnosis and Treatment of Severe Hepato-Pancreatic Diseases of Zhejiang Province, Zhejiang Provincial Top Key Discipline in Surgery, The First Affiliated Hospital of Wenzhou Medical University, Wenzhou, Zhejiang, China

## Abstract

**Background:**

Adipose-derived mesenchymal stem cells (ADMSCs) can promote healing and inhibit inflammation/immune response in local tissues, while the detailed mechanism remains unknown.

**Results:**

ADMSCs and peritoneal macrophages were collected from C57BL/6 mice. The culture medium (CM) from ADMSCs (24 hours cultured) was collected. The CM was added to the M*φ* culture system with lipopolysaccharide (LPS) or IL-4/IL-13 or blank. And those M*φ* cultures without adding CM were used as controls. A series of classification markers and signaling pathways for M*φ* polarization were detected by using flow cytometry, RT-PCR, and western blotting. Furthermore, the cell viability of all the groups was detected by CCK8 assay. After CM induction in different groups, M1-M*φ* markers and M2a-M*φ* were decreased; however, M2b/c-M*φ* markers increased. STAT3/SOCS3 and STAT6/IRF4 were suppressed in all 3 CM-treated groups. Moreover, the cell viability of all 3 groups which were induced by CM significantly increased as compared to that of the control groups without adding CM.

**Conclusion:**

ADMSCs can induce nonactivated macrophage and M1-M*φ* into M2b/c-M*φ*. Downregulation of the STAT3 and STAT6 pathway may involve in this process. This data shows that the anti-inflammatory role of ADMSC in local tissues may be partly due to their effect on M*φ* to M2b/c-M*φ*.

## 1. Introduction

Stem cells are characterized by their self-renewal ability and differentiation potential. Most adult stem cells are minor populations found in adult organs. They cannot give rise to an organism and only differentiate to specific cell lineages. Mesenchymal stem cells (MSCs) belong to this group, but some recent studies suggest that their paracrine action [1] and immunosuppressive properties [2] might be more meritorious than transdifferentiation. This phenomenon raises an intriguing question: What is the impact of MSC on the other cells in local tissues.

Resident tissue macrophages can rapidly respond to the microenvironment with the process of M*φ* activation. The classically activated M*φ* (M1-M*φ*) is generated in response to IFN-*γ* and TNF-*α* signal and resulted in the hypersecretion of IL-12. M1-M*φ* can be induced by bacteria or their products lipopolysaccharide (LPS). These cells kill intracellular pathogens and secrete a battery of inflammatory cytokines. In contrast, M2-M*φ* mainly involved in wound repair (M2a-M*φ*) and immune response (M2b/c-M*φ*) and M2a activation depends mainly on IL-4 and IL-13 [3].

The mononuclear phagocyte system is of hematopoietic origin, and a part of monocyte subsets differentiates into tissue macrophages [4]. Then, certain cytokines and microbial products will make a conversion in these cells from resting to more functionally active cells. These active cells can be divided into three types according to their function [5]: classically activated macrophages (M1-M*φ*), wound-healing macrophages (M2a-M*φ*), and regulatory macrophages (M2b/c-M*φ*). Different activated macrophages play a different role in local inflammation or systemic inflammation and multiple-organ dysfunction, which makes M*φ* valuable therapeutical targets [6]. The report on the transformation between M1 and M2-M*φ* shows this conception more realistic significance [7]. Meanwhile, the report on the MSC as salvage therapy for steroid-refractory acute graft-versus-host disease [8] shows that their paracrine action and immunosuppressive properties are highly recognized, suggesting the existence of the relationship between MSC and immune cells.

Based on the findings above, we used a series of approaches to explore the effect of ADMSC on activation of M*φ* and find out which signal pathway might be involved. This will help us to understand the role of ADMSC in immunosuppression in the local tissue.

## 2. Materials and Methods

### 2.1. Isolation of ADMSCs and Collection of the Culture Medium

Adipose tissue was obtained from 6 to 8-week-old C57BL/6 male mice (SLRC Laboratory Animal Company, Shanghai, China). Prior to the collection of the adipose tissue, mice were killed by cervical dislocation. The adipose tissue was excised from the iliac region and minced before digesting in DMEM (low glucose, Gibco-BRL, NY, USA) supplemented with 10% fetal bovine serum (FBS; Gibco-BRL, NY, USA) and 1 mg/ml collagenase type I solution (Sigma-Aldrich, St. Louis, MO, USA) in a vibration culturing box for 1 hour at 37°C. After 3 washings by centrifugation at 1200 rpm for 5 minutes in phosphate-buffered saline (PBS), cells were cultured (37°C, 5% CO_2_) in DMEM plus 10% FBS. After 24 hours of adhesion, nonadherent cells were removed and anchorage-dependent cells were cultured to passage 2 and reached 85% confluence. The medium was refreshed and collected after 24 hours, and cells were detected by flow cytometry. The collected culture medium (CM) was filtered with a 22 *μ*m filter to eliminate the cell debris.

### 2.2. Isolation, Activation, and CM Induction of Peritoneal Macrophages

Peritoneal macrophages were harvested by 5 peritoneal washes with 1 ml of PBS containing 3 U/ml heparin. The obtained cell suspension was centrifuged at 1200 rpm for 5 minutes. Cells were suspended in the RPMI 1640 culture medium containing 10% FBS and cultured (37°C, 5% CO_2_) for 8 hours. Then, the nonadherent cells were removed. The anchorage-dependent cells were detected by flow cytometry or activated and induced by the CM. The grouping of macrophages is detailed in Table 1. Cells were cultured (37°C, 5% CO2) in DMEM plus 10% FBS with reagents mentioned above; LPS (lipopolysaccharide), IL-4, and IL-13 were purchased from Sigma-Aldrich (St. Louis, MO, USA).

### 2.3. Real-Time PCR Analysis

Total cellular RNA was extracted from the different groups using the TRIzol reagent (Gibco-BRL). Subsequently, mRNA was reversely transcribed to single-stranded cDNA using the First-Strand cDNA Synthesis Kit (Thermo, Waltham, MA, USA). Real-time PCR was conducted with the ABI 7500 Thermal Cycler Dice™ real-time system using the SYBR® Green Real-time PCR Master Mix (Toyobo, Osaka, Japan). These primer pairs are presented in Table 2, partly referred to Edwards and colleagues' research [3]. For each sample, the relative mRNA expression levels were derived from the ratio of their expression to GAPDH expression as an internal standard using the 2^-ΔΔCt^ method.

### 2.4. Western Blot Analysis

M*φ* after activation and CM induction were harvested and lysed in lysis buffer containing protease and phosphatase inhibitors. The lysates were centrifuged at 12000 rpm for 15 min at 4°C. The supernatant was collected, and protein concentrations were determined by BCA protein assay. Proteins were subjected to SDS-PAGE analysis, transferred to nitrocellulose membranes, and incubated with the primary antibodies against STAT3, p-STAT3 (Cell Signaling Technology, MA, USA), IL10, IL27 R*α* (Abcam, Cambridge, UK), IL-12b p40, p-STAT6 (Santa Cruz Biotechnology, Santa Cruz, CA), GAPDH, IRF-4, and SOCS3 (Bioworld Technology, Nanjing, China). Protein bands were detected using an enhanced chemiluminescence reagent (Sigma-Aldrich, St. Louis, MO, USA) following incubation with horseradish peroxidase- (HRP-) conjugated goat anti-rabbit secondary antibodies (Bioworld Technology, Nanjing, China). The expression levels of the proteins were compared to the control based on the relative intensities of the bands.

### 2.5. Immunofluorescent Staining for Assessment of Cell Morphology

Different groups of M*φ* were washed with PBS and fixed in 4% paraformaldehyde for 10 min. After permeabilizing with PBS containing 0.3% Triton X-100 for 10 min and soaking in 10% goat serum (Solarbio, Beijing, China) with PBS at 37°C for 30 min, cells were incubated with the IL10, IL-12b p40, and IL27 R*α* antibody in PBS overnight. Subsequently, cells were then washed with PBS, and the secondary antibody solution mixed with PBS was incubated at 37°C for 1 hour in the dark room. After washing the cells three times with PBS, they were covered with DAPI working solution (Roche Science, Mannheim, Germany). Finally, they were examined using a DM4000B automated upright microscope system (Leica, Wetzlar, Germany).

### 2.6. Cytokinetics Analysis by CCK-8

M*φ* was plated in 96-well plates at 3 × 10^4^ cells/ml. Macrophages were activated according to the methods above. Subsequently, 100 *μ*l DMEM plus 10 *μ*l of CCK-8 (Dojin Chemicals Co., Kumamoto, Japan) were added to each well and incubated at 37°C for 30 min. The optical density (OD) of each well was then measured using the Varioskan microplate reader (Thermo, Waltham, MA, USA) at a 450 nm wavelength.

### 2.7. Flow Cytometry

ADMSCs were identified for surface markers by using CD90.2 and CD105 antibodies (eBioscience, San Diego, CA, USA). M*φ* was identified by using CD11b and F4/80 antibodies (eBioscience). M*φ* after activation and CM induction were stained using CD197 (eBioscience, CA, USA) and CD206 (BioLegend, CA, USA) at 4°C for 30 min. Cells were washed by PBS then detected by flow cytometry. All experiments were repeated three times.

### 2.8. Statistical Analysis

Statistical analysis was conducted using the SPSS software version 19.0. The analysis of variance (ANOVA) was used to test the difference in the means between groups. Differences were considered statistically significant at *P* < 0.05 (∗) or 0.01 (∗∗).

## 3. Results

### 3.1. Identification of Isolated ADMSCs and Macrophages

**Figure 1:**
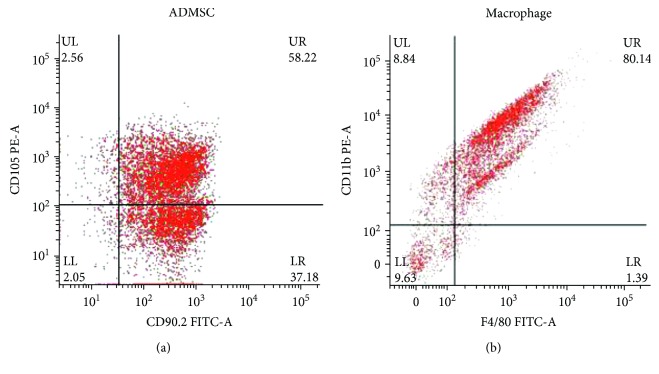
Flow cytometry analysis of two kinds of isolated cell surface antigen. CD105 and CD90.2 double positive cells of about 58.22% in isolated ADMSCs. CD11b and F4/80 double positive cells of about 80.14% in isolated M*φ*.

By using the flow cytometer, the isolated ADMSCs and macrophages were identified. CD105 and CD90.2 double positive cells, regarded as ADMSCs, are of about 58% (Figure 1(a)); CD11b and F4/80 double positive cells, regarded as macrophages, are of about 80% (Figure 1(b)).

### 3.2. Morphology of Induced M*φ*

**Figure 2:**
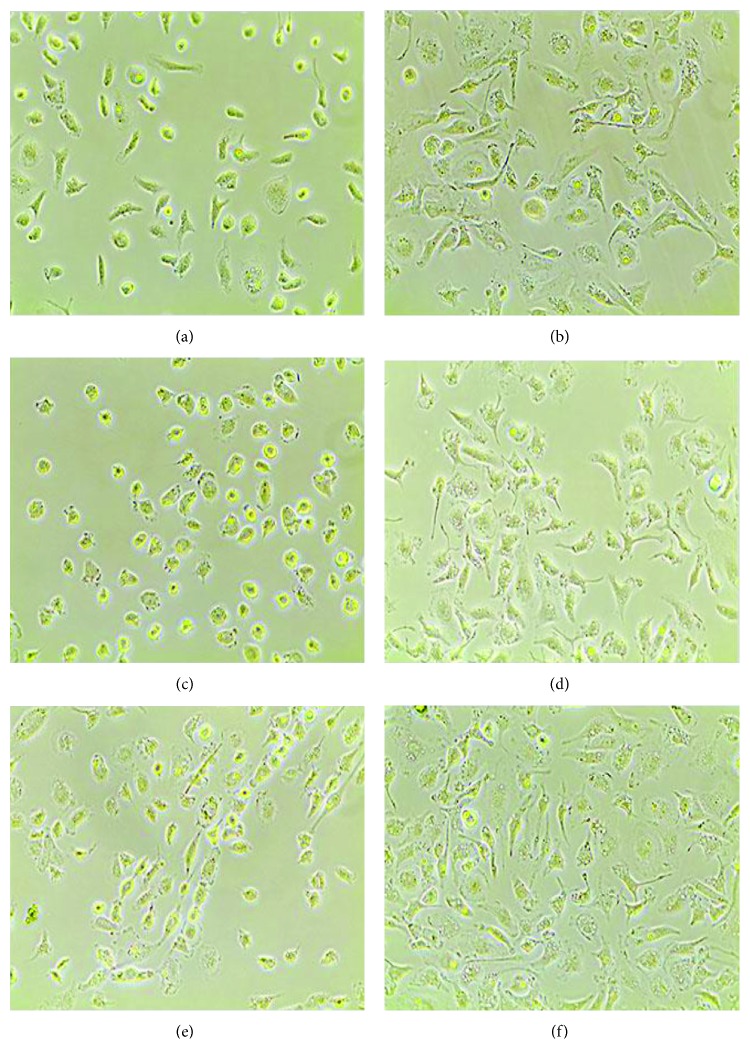
(a) Control group, (b) CM group, (c) LPS group, (d) LPS-CM group, (e) IL group, and (f) IL-CM group. M*φ* showed fried egg shapes after LPS induction in (c) or a spindle type after IL4/13 induction in (e). After ADMSC-CM induction, those fried egg-shaped or spindle-like cells were stretched with many cell membrane protuberances and a part of them became irregular (b, d, f).

The changes of M*φ* in morphology are obvious and stable. After LPS or IL4/13 induction, the morphological changes of M*φ* are consistent with the previous report [9]. Then, those cells were stretched with many cell membrane protuberances, and a part of them became irregular, following ADMSC-CM induction (Figure 2). These changes imply the activation of M2-M*φ* by ADMSC-CM.

### 3.3. Flow Cytometry

**Figure 3:**
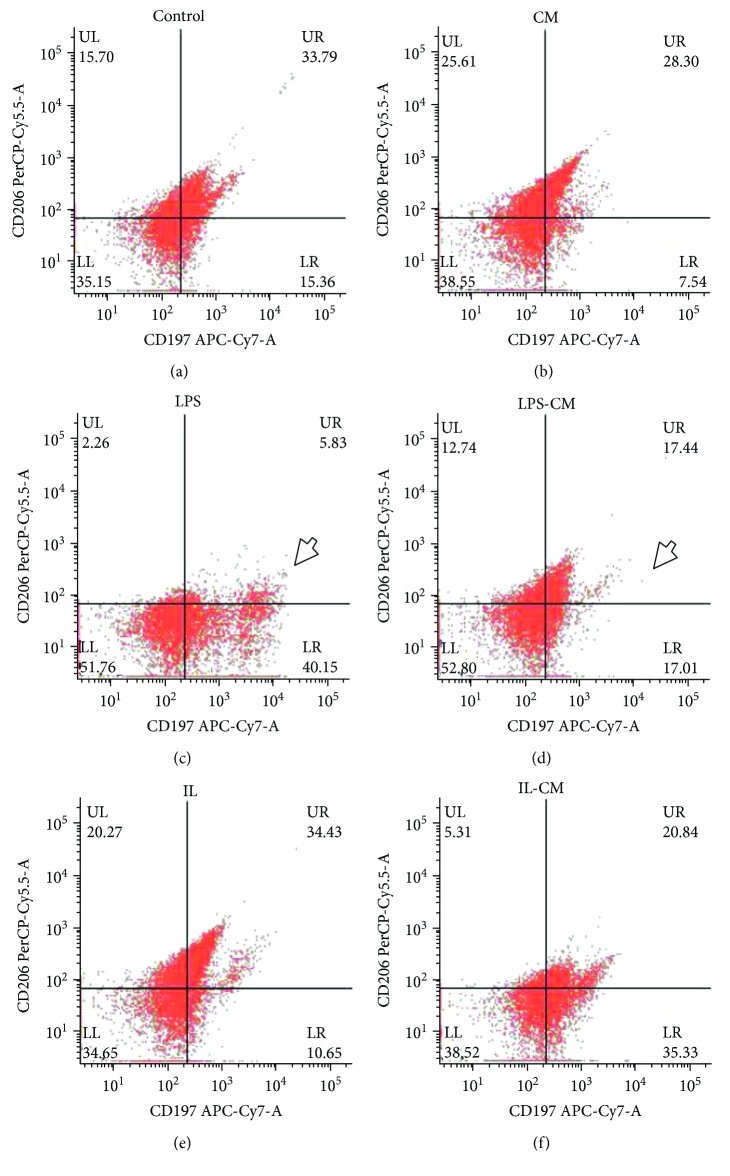
Flow cytometry analysis of different groups of M*φ*. Compared with the other groups, after LPS induction, the fluorescence intensity of CD206 in M*φ* decreased and obviously, there was a group of independent CD197^+^ M*φ* (c). This group of CD197^+^ M*φ* disappeared after ADMSC-CM induction (d).

Flow cytometry was also used to observe the effects of the ADMSC on macrophage. After LPS induction, a part of peritoneal macrophages showed the appearance of the CD197 positive which is an important marker of M1-type macrophages. Then, the CD197+M*φ* was significantly decreased following ADMSC-CM induction (Figures 3(c) and 3(d)). There was no significant difference between CD197+ M*φ* in the non-CM-induced groups and the control group. M*φ* induced by ADMSC-CM and IL-4/13 have no difference in the expression of CD197 or CD206.

**Figure 4:**
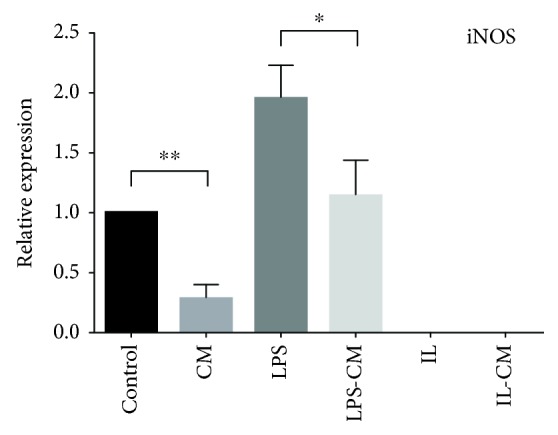
iNOS mRNA relative expression in different groups of M*φ*. Nonactivated M*φ* upregulate mRNA expression of iNOS after LPS induction. Both the nonactivated M*φ* and the LPS-induced M1-M*φ* downregulate mRNA expression of iNOS after ADMSC-CM induction. The fluorescence intensity of the IL and IL-CM groups does not exceed the fluorescence threshold in 40 cycles in RT-PCR.

**Figure 5:**
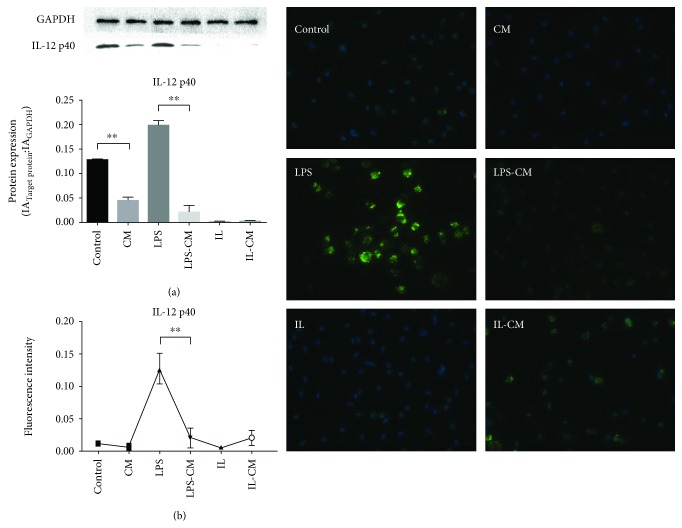
IL-12 p40 expression in different groups of M*φ*. (a) WB and its gray relative analysis. Nonactivated M*φ* upregulate the expression of IL-12 p40 after LPS induction. Both the nonactivated M*φ* and the LPS-induced M1-M*φ* downregulate the expression of IL-12 p40 after ADMSC-CM induction. The expression of IL-12 p40 in the IL and IL-CM groups was very low. (b) The relative fluorescence intensity analysis. The expression of IL-12 was effectively reduced after ADMSC induction. The change of fluorescence intensity of other groups was not obvious.

Meanwhile, killing function of macrophage depends heavily on nitric oxide. Upregulated mRNA expression of iNOS was found in LPS-induced M1 from nonactivated M*φ*. After ADMSC-CM induction, the downregulation of mRNA expression of iNOS both in the nonactivated M*φ* and the LPS-induced M1-M*φ* was detected (Figure 4). IL-12 is an important marker links to M1 activation of macrophage. And WB was used to detect the expression of IL-12. The trend of the expression of IL-12 was same to that of iNOS mRNA in each group (Figure 5). At the same time, immunofluorescence results also showed that the expression of IL-12 was effectively reduced after ADMSC-CM induction. The change of fluorescence intensity of non-ADMSC-CM-induced groups was not obvious.

### 3.4. ADMSC-Induced M2b/c-M*φ*

**Figure 6:**
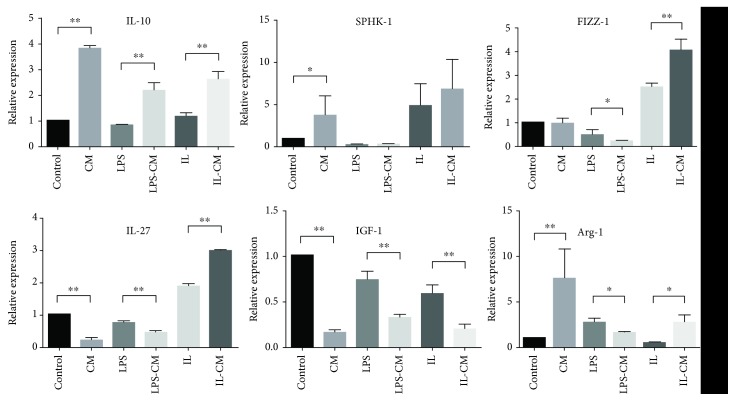
The mRNA expression in different groups of M*φ*. IL-10 and SPHK-1 are M2b/c markers. IL-27, IGF-1, FIZZ-1, and Arg-1 are M2a markers. Compared with the control, LPS, and IL groups, M*φ* in the CM, LPS-CM, and IL-CM groups upregulated their M2b/c marker mRNA expression, except SPHK-1 mRNA expression of the LPS-CM group. Compared with the control and LPS groups, M*φ* in the CM and LPS-CM groups downregulated their M2a markers mRNA expression, except FIZZ1-1 and Arg-1 mRNA expression of the control group. The IL-CM group upregulated their M2a marker mRNA expression, except IGF-1, compared with the IL group.

According to the macrophage classification standard of Mosser and Edwards [5], we also tested a series of M2 markers. The mRNA expression of IL-10 and SPHK-1, as M2b/c activation markers, was upregulated following ADMSC-CM induction in nonactivated M*φ* and IL-4/13-induced M*φ* (M2). IL-10 mRNA expression in LPS-induced M1-M*φ* was upregulated following ADMSC-CM induction (Figure 6).

The mRNA expression of M2a markers, including IL-27, IGF-1, FIZZ-1, and Arg-1, in LPS-induced M1-M*φ*, was downregulated following ADMSC-CM induction. IL-27 and IGF-1 mRNA expression in nonactivated M*φ* was downregulated following ADMSC-CM induction, while the Arg-1 expression was upregulated. More intriguingly, after addition of ADMSC, IL-4/13-induced M2-M*φ* upregulated FIZZ-1, IL-27, and Arg-1 mRNA expression. Only IGF-1 was downregulated.

**Figure 7:**
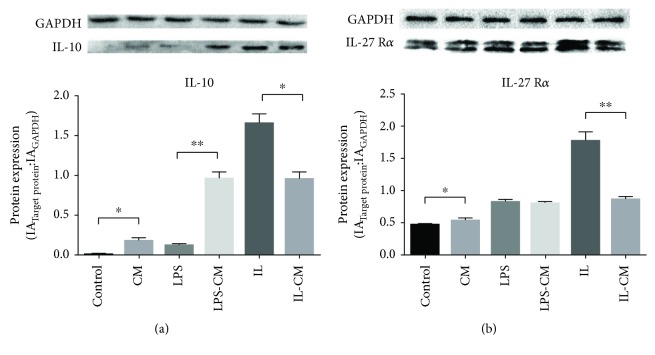
IL-10 and IL-27 expression in different groups of M*φ*. IL-10 is highly expressed in the LPS-CM, IL, and IL-CM groups. Compared with the control and LPS groups, CM and LPS-CM increased their IL-10 expression, after ADMSC induction. But IL4/13-induced M2-M*φ* decreased IL-10 expression, after ADMSC induction. On the contrary, IL-27 is highly expressed in IL4/13-induced M2-M*φ* and was decreased after ADMSC induction.

The expression of IL-10 and IL-27 R*α* was detected by WB. The expression of IL-10 in LPS-CM, IL, and IL-CM groups was higher than other control groups. After ADMSC induction, nonactivated M*φ* and LPS-induced M1-M*φ* increased their IL-10 expression (Figure 7(a)). On the contrary, IL4/13-induced M2-M*φ* downregulated IL-10 expression after ADMSC induction. IL-27 R*α* is highly expressed in IL4/13-induced M2-M*φ* and was decreased after ADMSC induction (Figure 7(b)).

### 3.5. Effects of ADMSC on the Macrophage Jak/STAT Pathway

**Figure 8:**
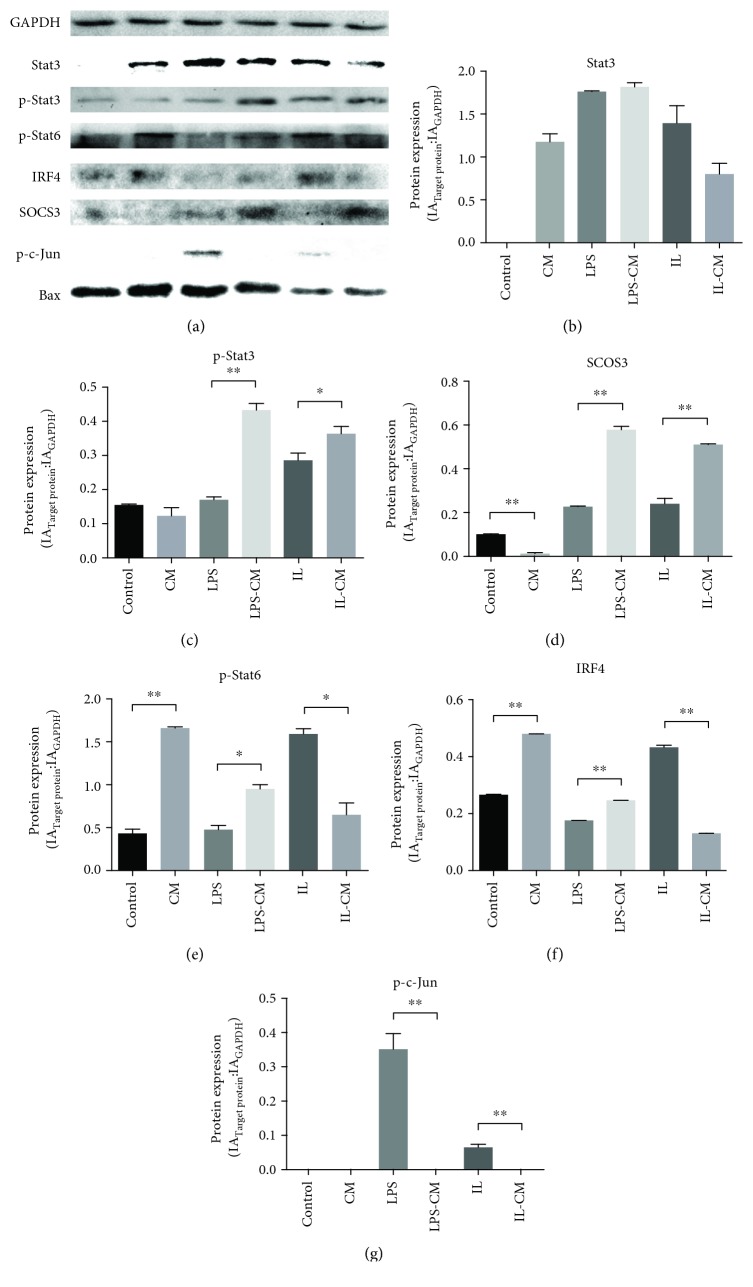
The relative expression of a series of pathway proteins in different groups of macrophages. After ADMSC induction, nonactivated M*φ* and LPS-induced M1-M*φ* increased their expression of p-STAT6 and its downstream IRF4 (d and e). On the contrary, IL-4/13-induced M2-M*φ* decreased their expression after ADMSC induction. On the other hand, after ADMSC induction, LPS-induced M1-M*φ* and IL-4/13-induced M2-M*φ* increased their expression of p-STAT3 and its downstream SOCS3 (b and c). The expression of p-STAT3 in nonactivated M*φ* showed no significant change after ADMSC induction, while the expression of SOCS3 decreased.

The Jak/STAT pathway is one of the most important pathways of the macrophage activation process. After ADMSC induction, nonactivated M*φ* and LPS-induced M1-M*φ* upregulated their expression of p-STAT6 and its downstream IRF4 (Figures 8(d) and 8(e)). On the contrary, IL-4/13-induced M2-M*φ* decreased p-STAT6/IRF4 expression after ADMSC induction. On the other hand, after ADMSC induction, LPS-induced M1-M*φ* and IL-4/13-induced M2-M*φ* increased their expression of p-STAT3 and its downstream SOCS3 (Figures 8(b) and 8(c)). The expression of p-STAT3 in nonactivated M*φ* showed no significant change after ADMSC induction, while the expression of SOCS3 decreased.

The JNK pathway, which can be activated by LPS, can phosphorylate STAT1 and cause M1 activation. As an important indicator of the JNK pathway, p-c-Jun is highly expressed in LPS-induced M1-M*φ*, slightly expressed in IL-4/13-induced M2-M*φ*, and almost not expressed in nonactivated M*φ* and other groups which were induced by ADMSC-CM (Figure 8(g)). Therefore, ADMSC has an obvious inhibitory effect on the JNK pathway.

### 3.6. Viability of M*φ* following ADMSC Induction

Macrophages have very strong adherence ability. As shown in Table 3, new isolated M*φ* exist in 0.25% trypsin for 30 minutes and half of the cells remain adherent. But after 4 days of culture in vitro, nonactivated M*φ* became low adherent. The LPS-induced M*φ* group became even lower, and a large number of floating cells and cell debris appeared. On the contrary, M*φ* in the LPS-CM group adhered more tightly than the control and LPS groups. And, M*φ* in the CM, IL, and IL-CM groups has a strong adhesion as new isolated M*φ*. This means that M*φ* in the CM, LPS-CM, IL, and IL-CM groups are in good condition after 4 days of culture in vitro.

**Figure 9:**
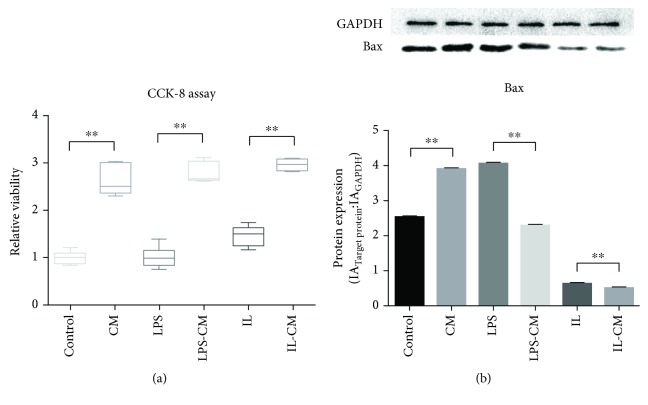
CCK-8 assay and Bax expression of a series of pathway proteins in different groups of macrophages. (a) The relative viability of all groups. The relative viability of all the groups which were induced by ADMSC increased. (b) Bax expression of groups. Bax expression in the IL and IL-CM groups was decreased.

Based on the above phenomenon, M*φ* viability and Bax expression were detected. The results show that IL-4 and IL-13 can decrease the expression of Bax, while MSC can improve the cell activity of macrophages. The cell viability of all the groups which were induced by ADMSC-CM increased based on the CCK8 assay, while M*φ* in the IL and IL-CM groups downregulated the expression of Bax (Figure 9).

## 4. Discussion

### 4.1. M1-M*φ* Induced to M2-M*φ* by ADMSC

Induction to polarization of macrophages is a potential method of the treatment of inflammatory injury. MSC can secrete a variety of cytokines [10]; we wondered if these cytokines could affect the activation of macrophages. For nonactivated M*φ* and M1-M*φ*, our data shows that ADMSC induction can decrease the M1 marker expression (iNOS and IL-12) and increase the M2b/c marker expression (IL-10 and SPHK-1). Previous reports show that MSCs directly inhibit inflammation by promoting macrophage polarization toward M2 [11, 12]. Our results further confirmed that M2-M*φ* markers especially M2b/c increased significantly after adding ADMSC to LPS-induced M1-M*φ*. This shows that ADMSC can inhibit the differentiation to M1-M*φ* and induce the nonactivated M*φ*/M1-M*φ* to M2-M*φ*.

M1-M*φ* is involved in cell-mediated immune response and accomplished during infection by producing a series of proinflammatory cytokines, which also lead to host-tissue damage. M2-M*φ* with high phenotypic heterogeneity produced anti-inflammatory cytokines to attenuate inflammation [13]. After ADMSC induction, M1-M*φ* downregulated the expression of p-c-Jun and iNOS and upregulated the expression of p-Stat3, p-Stat6, and IL-10. ADMSC induced M1-M*φ* to M2-M*φ* suggests that ADMSC directly regulates direction of macrophage polarization to participate in the inflammatory process.

### 4.2. ADMSC Induced “Not Fully Activated M2-M*φ*” Similar to M2b/c-M*φ*

M2 includes M2a, M2b, and M2c three subtypes each with different functions in the inflammation process [14]. However, the type of M2-M*φ* induced by ADMSC is complex. At mRNA levels, all the M2a-M*φ* markers still keep upregulated after adding CM; moreover, M2b/c markers were upregulated too. At the protein level, the expression of IL-10 and IL-27 decreased after addition of CM. M*φ* seem to be induced to a kind of M2-like M*φ*, which have an independent set point.

A previous research showed that the JNK pathway is associated with LPS-induced M1 activation, while the Jak/STAT6 pathway is mainly links to IL-4/13-induced M2 activation [15]. And, the Jak/STAT3 pathway is related to both of them [16, 17]. Our data shows that ADMSC induction could activate the expression of STAT6 and its downstream IRF4 in nonactivated M*φ* and M1-M*φ*. Moreover, the expression of IL-10 also increased in both types of M*φ*. This evidenced that the effects of ADMSC on nonactivated M*φ* and M1-M*φ* are reliable. However, ADMSC-induced M2-M*φ* was different from IL-4/13-induced M2-M*φ*; p-STAT6 and IRF-4 were downregulated in IL-4/13-induced M2-Mϕ but upregulated in ADMSC-induced M2-Mϕ as shown in (Figure 8). This data verifies our hypothesis that ADMSC-induced M2 is not a kind of “not fully activated M2-M*φ*”, but a kind of M2-like M*φ* with its own set point.

As a proinflammatory signaling pathway, the activation of JNK signaling facilitates a high expression of proinflammatory factors such as NO, TNF-*α*, and IL-1*β* [18]. Our data shows that p-c-Jun is highly expressed in M1-M*φ* and slightly expressed in M2-M*φ*, but almost no expression in nonactivated M*φ* and all ADMSC-induced M*φ*. Lower expression of p-c-Jun indicates lower inflammatory response of ADMSC-induced M*φ*. Considering the protein expression of IL-10 and IL-27, ADMSC-induced M*φ* also was different from classic IL-4/13-induced M2-M*φ*. This is another reason for not considering ADMSC-induced M*φ* as a kind of “not fully activated M2-M*φ*”.

Compared with M2a-M*φ*, ADMSC-induced M*φ* was more similar to M2b/c-M*φ*, which is a potent cell type of inhibiting inflammation. The most prominent feature of M2b/c-M*φ* is the secretion of a large number of IL-10, which can inhibit the production and activity of various proinflammatory cytokines [13, 19]. Consistent with this, our results showed that all of the ADMSC-induced macrophages upregulated the mRNA expression of IL-10. Although M2b/c-M*φ* can produce many proinflammatory cytokines, the production of IL-10 let M2b/c-M*φ* show the main function of inhibiting inflammation.

### 4.3. Potential ADMSC-Secreted Cytokine Induced M2b/c-M*φ*

**Figure 10:**
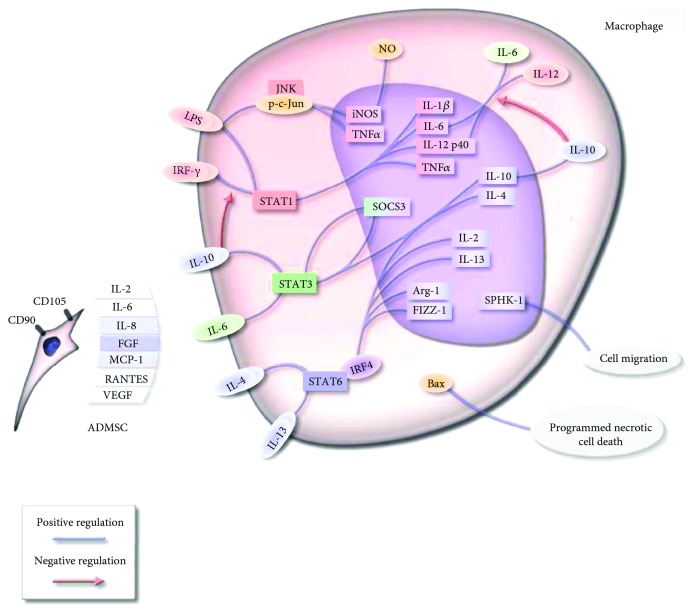
A work model for induction of macrophage M2b/c polarization by adipose tissue-derived mesenchymal stem cells.

Previous studies indicate that ADMSC secrete a couple of cytokines including IL-2, IL-6, IL-8, FGF, MCP-1, RANTES, and VEGF (Figure 10) [20, 21]. Among these, IL-6 is a major cytokine and exerts activity by regulating JAK/STAT signal transduction [20–22]. Our data confirmed the obvious change of the JAK/STAT pathway in ADMSC-induced macrophage (Figure 8). Moreover, the M2b activation is mainly a response to IL-6 accompanying high expression of IL-10 [13, 20]. Our results found the high expression of IL-10 in ADMSC-induced macrophage. Furthermore, the regulation on macrophage polarization is induced by multiple cytokines secreted by ADMSC. For example, IL-8 secreted by MSC induced macrophage to M2 macrophage and VEGF probably affect transformation of M1 macrophages into M2 macrophages [23, 24]. Considering the mentioned above, the polarization of M*φ* to M2b/c-M*φ* may be induced by ADMSC-secreted cytokines especially IL-6.

The regulation of macrophage activation is one of the regulatory mechanisms of ADMSC downregulation of inflammation. In some severe inflammation, the activation of a large number of white blood cells, including macrophages, will release a lot of inflammatory cytokines [25]. Based on the phenomenon we observed, all the M2-M*φ* not only downregulated the inflammation but also prevent cell apoptosis and maintain a strong adherent ability. IL-4/13 or ADMSC-induced M*φ* decreased Bax expression, while increased cell viability. The combination of IL-4/13 and ADMSC has an important application value.

In summary, the anti-inflammatory role of ADMSC in local tissues may be due to their effect on M*φ* and induce them to M2b/c-M*φ*. Then, those M2b/c-M*φ* play a pivotal role in regulating the progression of the inflammatory process. This mechanism is a reasonable explanation for why ADMSC can treat immunological/inflammatory diseases, such as the allergic [26], rheumatoid [27, 28], and graft-versus-host diseases [8]. As a differentiated cell, ADMSC has a powerful regenerative function. But the immune regulation ability of ADMSC just like adding wings to a tiger greatly improves the application value of ADMSC (Figure 10).

## Figures and Tables

**Table 1:** The grouping of macrophages.

Group	0-24 h	25-72 h
Control	—	—
CM	—	ADMSC-CM (50%)
LPS	LPS (1 *μ*g/ml)	—
LPS-CM	LPS (1 *μ*g/ml)	ADMSC-CM (50%)
IL	IL-4, IL-13 (1 *μ*g/ml)	—
IL-CM	IL-4, IL-13 (1 *μ*g/ml)	ADMSC-CM (50%)

**Table 2:** Primers used in real-time PCR analysis.

Gene	Accession number	Primers
GAPDH	NC_000072.6	5 ′-AGGTCGGTGTGAACGGATTTG-3 ′
		5 ′-TGTAGACCATGTAGTTGAGGTCA-3 ′

Arg-1	NC_000076.6	5 ′-CAGAAGAATGGAAGAGTCAG-3 ′
		5 ′-CAGATATGCAGGGAGTCACC-3 ′

FIZZ1	NM_020509	5 ′-GGTCCCAGTGCATATGGATGAGACCATAGA-3 ′
		5 ′-CACCTCTTCACTGCAGGGACAGTTGGCAGA-3 ′

IGF-1	NC_000076.6	5 ′-CACACCTCTTCTACCTGGCGCTCTGC-3 ′
		5 ′-AGTCTCCTCAGATCACAGCTCCG-3 ′

IL-10	NC_000067.6	5 ′-CCAGTTTTACCTGGTAGAAGTGATG-3 ′
		5 ′-TGTCTAGGTCCTGGAGTCCAGCAGACTCAA-3 ′

IL-12 p40	NM_008352	5 ′-ATGGCCATGTGGGAGCTGGAGAAAG-3 ′
		5 ′-GTGGAGCAGCAGATGTGAGTGGCT-3 ′

IL-27 p28	NC_000073.6	5 ′-GGCCATGAGGCTGGATCTC-3 ′
		5 ′-AACATTTGAATCCTGCAGCCA-3 ′

iNOS	NM_010927	5 ′-CCCTTCCGAAGTTTCTGGCAGCAGC-3 ′
		5 ′-GGCTGTCAGAGCCTCGTGGCTTTGG-3 ′

SPHK1	NM_011451	5 ′-ACAGCAGTGTGCAGTTGATGA-3 ′
		5 ′-GGCAGTCATGTCCGGTGATG-3 ′

TNF*α*	NC_000083.6	5 ′-TTAACGCCCCACTCACCTGCTG-3 ′
		5 ′-GCTTCTTTGGGACACCTGCTGC-3 ′

**Table 3:** Adherence of M*φ* cultured in vitro for 4 days.

Attachment efficiency	In culture medium	In 0.25% trypsin		
		5-10 min	20 min	30-40 min
Control	80-90%	<10%		
CM	>95%			>40%
LPS	70-80%	<10%		
LPS-CM	90-95%	<40%	<10%	
IL	>95%			>40%
IL-CM	>95%			>40%

## Data Availability

The data used to support the finding of this study “Induction of Macrophage M2b/c Polarization by Adipose Tissue-Derived Mesenchymal Stem Cells” are included within the article.
